# Transpupillary Argon Laser Cyclophotocoagulation in a Refractory Traumatic Glaucoma Patient with Aphakia and Aniridia

**DOI:** 10.4274/tjo.22230

**Published:** 2016-01-05

**Authors:** Umut Duygu Uzunel, Berna Yüce, Tuncay Küsbeci, Halil Ateş

**Affiliations:** 1 İzmir Education and Research Hospital, Ophthalmology Clinic, İzmir, Turkey; 2 Giresun University Faculty of Medicine, Department of Ophthalmology, Giresun, Turkey; 3 Ege University Faculty of Medicine, Department of Ophthalmology, İzmir, Turkey

**Keywords:** Transpupillary argon laser cyclophotocoagulation, traumatic aniridia, aphakia, glaucoma

## Abstract

We present a case of transpupillary argon laser cyclophotocoagulation (TALC) in a patient with traumatic aniridia and aphakia secondary to blunt trauma who had previous bilateral trabeculectomy. Four months after the trauma the patient’s intraocular pressure (IOP) rose to 35 mmHg despite topical antiglaucomatous medication. Inferior 180 degrees cyclophotocoagulation was performed with transpupillary argon laser in the first session and his IOP fell to values of 12-17 mmHg. Twelve weeks after TALC, his IOP rose to 22 mmHg and we had to apply TALC to the residual ciliary processes. Seven months later his IOP was 13 mmHg with topical dorzolamide/timolol and latanoprost administration. TALC may be an effective treatment alternative for lowering IOP in patients with visible ciliary processes who do not respond to conventional medical or laser treatment.

## INTRODUCTION

Transpupillary argon laser cyclophotocoagulation (TALC) is an alternative cyclodestructive procedure in selected patients with glaucoma.^1,2,3^ This procedure includes argon laser photocoagulation of the ciliary processes after visualization with a goniolens. The proportion of visualized ciliary processes depends on the extent of iris defect, which ranges from peripheral iridectomy to aniridia.

## CASE REPORT

**Figure 1:**
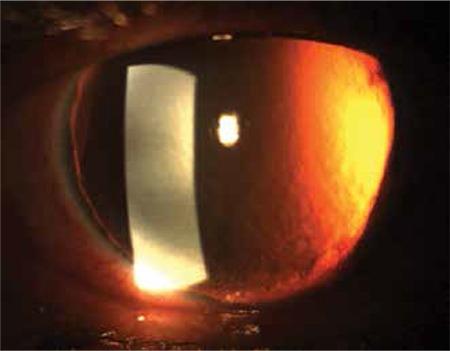
Anterior segment photograph of the right eye showing the aniridia and aphakia

**Figure 2:**
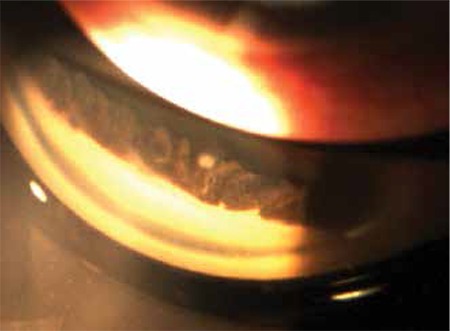
Gonioscopic photograph of the right eye showing the ciliary processes before transpupillary argon laser cyclophotocoagulation

A 55-year-old man who had been followed up at our glaucoma unit for 5 years with the diagnosis of primary open-angle glaucoma (POAG) presented with sudden loss of vision and pain in his right eye after a blunt trauma sustained from a fist during a physical confrontation. The patient had a history of bilateral trabeculectomy 2 years earlier. On initial examination after the blunt trauma to the right eye, visual acuity was light perception and the eye had a corneoscleral laceration approximately 5 mm long extending from the previous trabeculectomy incision. The iris was totally dialyzed and prolapsed from the wound along with the crystalline lens. After removal of the totally prolapsed iris and extruded crystalline lens, anterior vitrectomy and repair of the corneoscleral laceration with 10-0 nylon suture were performed. The intraocular pressure (IOP) was between 12-18 mmHg with topical anti-glaucomatous medication for a period of 4 months after the trauma. His IOP rose to 35 mmHg despite administration of topical latanoprost, dorzolamide/timolol fixed combination and brimonidine in the 4th month. Uncorrected visual acuity (UCVA) was hand motion, best corrected visual acuity (BCVA) was counting fingers from one meter with aphakic spectacle correction in the right eye and 20/20 in the left eye. IOP in the left eye was 13 mmHg with latanoprost and dorzolamide/timolol fixed combination. Clear cornea, quiet anterior chamber, aphakia and total aniridia were seen on slit-lamp examination of the right eye (Figure 1). Dilated fundus examination of the right eye revealed a normal retina and a glaucomatous optic nerve head with a cup-to-disc ratio of 0.9. Slit-lamp examination was normal and the cup-to-disc ratio was 0.7 in the left eye. The ciliary body was normal except the superior degenerated area of 60 degrees in the right eye (Figure 2). Surgical intervention (trabeculectomy with antimetabolite or tube shunt implantation) was planned to reduce the IOP. Complications of glaucoma surgery were explained to the patient, but we had to take into account alternative IOP reducing procedures due to patient’s refusal of any surgical intervention. Therefore, we planned TALC for the right eye to lower IOP.

A Goldmann three-mirror lens was placed onto the right eye after instillation of 0.5% proparacaine hydrochloride (Alcaine, Fort Worth, Texas, USA) in the laser therapy room. Methylcellulose was placed on the contact surface of the lens to fill the lens-cornea interface. The argon laser settings were 700 mW power, 100 µm spot size, and 0.1 seconds exposure time (Visulas 532s, Carl Zeiss Meditec AG, Jena, Germany). A total of 145 laser exposures were administered through the Goldmann contact lens to the inferior 180 degrees of the ciliary processes. We avoided performing 360-degree argon laser cyclophotocoagulation of the right eye due to the risk of phthisis bulbi. The patient did not report any remarkable pain or discomfort during laser treatment.

The patient was treated with dorzolamide/timolol fixed combination, latanoprost and topical ketorolac tromethamine after TALC. The anterior chamber was quiet, despite the lack of steroid treatment. The IOP ranged between 12-17 mmHg during the first 12 weeks after TALC. In the 12th week, IOP raised to 22 mmHg, so we performed TALC to the residual healthy ciliary processes of the right eye. A total of 105 exposures were administered using the same settings specified above.

**Figure 3:**
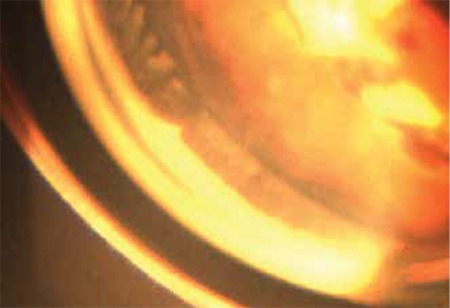
Gonioscopic photograph of same region of showing the ciliary processes 7 months after transpupillary argon laser cyclophotocoagulation

Seven months after the TALC procedure, the patient’s UCVA was counting fingers from one meter, BCVA was 20/200 with aphakic contact lens correction, and the diurnal mean IOP was 13 mmHg with topical dorzolamide/timolol and latanoprost administration. The ciliary processes were seen as atrophic on gonioscopy (Figure 3).

## DISCUSSION

Refractory glaucoma is a difficult condition to manage. In cases who are unresponsive to medical, laser, and surgical treatments for lowering IOP, drainage procedures, such as trabeculectomy with antimetabolite, are potential solutions but may be associated with a series of complications including hypotony, leaking blebs, and endophthalmitis.^4^ Bloom et al.^5^ showed that tube surgery, Nd-YAG laser, and diode laser cyclophotocoagulation all effectively lower IOP in the short and medium term in refractory glaucoma. They also reported that tube surgery was associated with a greater incidence of sight-threatening complications, despite its better control of IOP in refractory glaucoma. Kaplowitz et al.^6^ reported in their review that the visual outcomes were better with endoscopic cyclophotocoagulation (ECP) when compared with both trabeculectomy and aqueous shunt implantation, but the IOP outcomes were very similar. They concluded that ECP as a very effective and safe option in cases with refractory glaucoma.

When done as an outpatient procedure, TALC of the ciliary processes also shows promise as a convenient, low-risk, and useful alternative procedure in selected aphakic glaucoma cases that are poorly controlled by medical or surgical measures. Kim and Moster1 reported a case who had a significant decrease in IOP 10 weeks after TALC. Shields et al.^7^ reported a successful outcome in 6 of 27 patients. Merritt^8^ reported that only one in seven patients had a significant decrease in IOP after TALC and that patient had the largest proportion of their ciliary processes treated in the series. The author concluded that the limiting factor in effective TALC may be the total number of ciliary processes visualized and treated.^8^ In our case, the IOP lowering effect was limited when TALC was applied to only 180 degrees of the ciliary processes. The IOP lowering effect was increased when TALC was applied to all healthy ciliary processes. We did not observe any inflammatory reaction, so we believe that TALC is a repeatable procedure without any serious side effects.

## CONCLUSION

Aphakia may negatively affect the success rate of penetrating glaucoma surgery or tube shunt implantation due to blockage of the new drainage route by the vitreous. Thus, treatment alternatives that aim to reduce aqueous humour production may be chosen primarily in aphakic patients. TALC may be an effective treatment alternative for lowering IOP in patients with visible ciliary processes who do not respond to conventional medical or laser treatment. This approach may also be used as an adjunct to the medical and/or surgical management of selected glaucoma cases with aniridia and aphakia. TALC is a treatment which can be done under topical anesthesia and does not cause serious inflammation, so it may help physicians gain time to select the appropriate treatment for the patient. Future studies with large case series may shed more light on the advantages and limitations of this procedure.

## Ethics

Informed Consent: It was taken.

Peer-review: Externally peer-reviewed.
